# Solid‐State Far‐Ultraviolet C Light Sources for the Disinfection of Pathogenic Microorganisms Using Graphene Nanostructure Field Emitters

**DOI:** 10.1002/gch2.202200236

**Published:** 2023-03-06

**Authors:** Yoichiro Neo, Gai Hashimoto, Rei Koike, Takashi Ohhara, Takahiro Matsumoto

**Affiliations:** ^1^ Research Institute of Electronics Shizuoka University Hamamatsu 432‐8011 Japan; ^2^ Neutron Science Section J‐PARC Center Japan Atomic Energy Agency Ibaraki 319‐1195 Japan; ^3^ Graduate School of Medical Sciences Nagoya City University Nagoya 467‐8601 Japan; ^4^ Graduate School of Design and Architecture Nagoya City University Nagoya 464‐0083 Japan

**Keywords:** cathodoluminescence, disinfection, *Escherichia coli*, far ultraviolet, field emission, graphene

## Abstract

The ongoing global outbreak of coronavirus disease has necessitated the use of ultraviolet (UV) disinfection techniques to reduce viral transmission in public places. The previously used UV wavelength is harmful to the human body, the wavelength range from 200 to 235 nm, often referred to as far‐UVC light, has attracted attention as a novel disinfection wavelength range that can be used in a safe manner. However, the currently used light sources have practical problems, such as an expensive cost, a low efficiency, and short lifetimes. Therefore, environmentally friendly solid‐state light sources with a lower cost, higher efficiency, and longer lifetimes are demanded. Here, an efficient mercury‐free far‐UVC solid‐state light source is presented. This light source demonstrates intense 230 nm emission with a narrow spectral width of 30 nm and a long lifetime of more than 1000 h. These characteristics can be achieved by graphene nanostructure field emitters and wide‐bandgap magnesium aluminate phosphors. By using this light source, the efficient disinfection of *Escherichia coli* is demonstrated. The light sources presented here facilitate future technologies for preventing the spread of infectious diseases in a safe and convenient manner.

## Graphical abstract

The ongoing global outbreak of pathogenic viruses have necessitated the use of ultraviolet disinfection techniques to reduce viral transmission in public places. A highly efficient mercury‐free far‐UVC solid‐state light source is presented. This light source demonstrates intense 230 nm emission and long lifetime more than 1000 h. By using the light sources, the efficient disinfection of *Escherichia coli* is demonstrated.

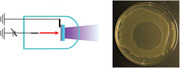

## 1 Introduction

With the global pandemic of severe acute respiratory syndrome coronavirus 2 (SARS‐CoV‐2), there is a strong demand to develop and demonstrate efficient disinfection techniques to protect against various pathogenic viruses and bacteria.^[^ ^1^ ^]^ The recent pandemic agent SARS‐CoV‐2, the causal agent of coronavirus disease 2019 (COVID‐19), is not only transmitted through respiratory droplets but can also spread through nasal, oral, and eye secretion‐contaminated surfaces.^[^ ^2^, ^3^ ^]^ Although vaccines provide effective protection against SARS‐CoV‐2 infection, the efficacy and supply speed of these vaccines against future emerging SARS‐CoV‐2 variants are not clear at the present stage.^[^ ^4^ ^]^ Therefore, it is important to prepare additional strategies against emerging pathogens to mitigate public health risks during the prevaccine development period.

Disinfection by ultraviolet (UV) irradiation is attracting special interest in regard to reducing SARS‐CoV‐2 transmission because UV irradiation offers an effective and convenient method for the inactivation of pathogenic microorganisms, including SARS‐CoV‐2.^[^ ^5^, ^6^, ^7^, ^8^, ^9^, ^10^ ^]^ In particular, the wavelength range from 200 to 235 nm, often referred to as far‐UVC light, has attracted increasing attention as a novel disinfection wavelength range. Because far‐UVC light has a strong germicidal effect on pathogenic viruses and bacteria^[^ ^11^, ^12^, ^13^, ^14^, ^15^ ^]^ and has been shown to be harmless to mammalian cells due to the strong absorption effect of the stratum corneum layer.^[^ ^16^, ^17^, ^18^, ^19^, ^20^ ^]^ However, currently used far‐UVC light sources such as 222 nm excimer lamps have many practical problems, such as an expensive cost of more than $1000, low power efficiency of less than 1%, and short lifetimes of the order of 1000 h. Therefore, environmentally friendly solid‐state light sources with a lower cost, higher efficiency, and longer lifetimes are strongly desired.

Aluminum–gallium nitride (AlGaN) UV light‐emitting diodes (LEDs) can be candidates to realize practical solid‐state far‐UVC devices, because the UV emission can be tuned to cover almost the entire UV spectrum from 210 to 365 nm by changing the stoichiometry between Al and Ga for AlGaN alloying materials.^[^ ^21^, ^22^, ^23^, ^24^ ^]^ Since the report of 210 nm emission by Taniyasu et al.,^[^ ^25^ ^]^ there have been many efforts to increase the output power and emission efficiency as well as to reduce the LED chip cost of AlGaN‐based LEDs in the far UVC region (210–235 nm).^[^ ^26^, ^27^, ^28^, ^29^ ^]^ However, the output power and emission efficiency are still limited because of several inherent issues, such as i) low hole concentrations and ii) high dislocation densities. To date, the quantum efficiency reported for far‐UVC LEDs (210–235 nm) is below 1%,^[^ ^25^, ^28^ ^]^ and this fact shows the difficulty of obtaining far‐UVC LEDs in practice.

Electron beam (EB)‐pumped far‐UVC light sources have been explored as a promising alternative to avoid these problems. Several groups have reported EB‐pumped far‐UVC light sources based on wide‐bandgap materials, such as hexagonal boron nitride powder,^[^ ^30^ ^]^ κ‐Al_2_O_3_ crystals^[^ ^31^, ^32^ ^]^ and ultrathin GaN/AlN multiple quantum wells.^[^ ^33^, ^34^ ^]^ Among these reports, a study by Oto et al.^[^ ^35^ ^]^ has attracted special interest regarding the possibility of EB‐pumped AlGaN quantum wells because the UV emission at the peak wavelength below 240 nm was obtained with a high output power of 100 mW and a high power efficiency exceeding 40%. The physical mechanisms of this efficient cathodoluminescence (CL) have not yet been clarified; nevertheless, this report broadens the possible applications of AlGaN from electron and hole injection to vacuum nanoelectronics.

In this work, a solid‐state mercury‐free far‐UVC light source is successfully attained based on vacuum nanoelectronics technologies. Herein, this light source demonstrates intense 230 nm emission with a narrow spectral width of 30 nm (full width at half maximum, FWHM) and long lifetime more than 1000 h. These characteristics can be obtained by using graphene nanostructure (GN) field emitters and wide‐bandgap magnesium aluminate (MgAl_2_O_4_) phosphors. Based on the far‐UVC light sources presented herein, we demonstrated the efficient disinfection of *Escherichia coli* (*E*. *coli*). The solid‐state far‐UVC light source proposed herein has many advantages, such as environmental friendliness, a long lifetime, a small size, and an affordable cost. Therefore, it is possible to apply this solid‐state light source in current lighting equipment. We believe that solid‐state far‐UVC light sources based on GN and wide‐bandgap phosphors will lead to future technology for preventing the spread of infectious diseases in a safe and convenient manner.

## 2 Results

### 2.1 Fabrication of a Solid‐State Far‐UVC Light Source

**Figure 1:**
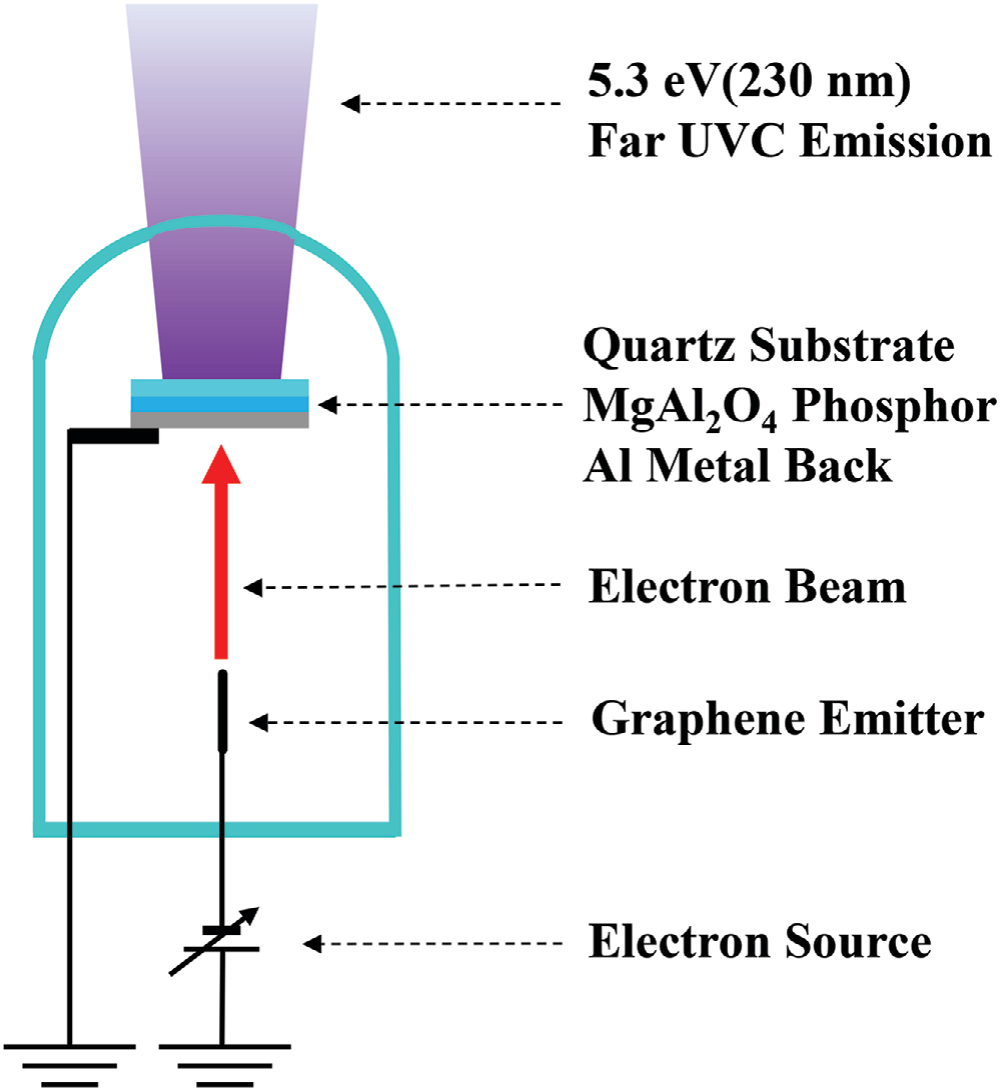
Solid‐state far‐UVC emission source structure. The device consists of a diode‐type field emission far‐UVC CL tube composed of a GN cold cathode and a MgAl_2_O_4_ phosphor screen anode. The electrons emitted from the GN cold cathode excite the phosphor, causing far‐UVC CL.

**Figure** **1** shows our far‐UVC emitting device structure. The device consists of a diode‐type field emission far‐UVC CL tube composed of a GN cold cathode and a MgAl_2_O_4_ phosphor screen anode. For the far‐UVC emission, a negative voltage of 3–25 keV was applied to the cathode. Electrons accelerated at this voltage were irradiated onto the phosphor anode; thus, it is possible to obtain efficient far‐UVC luminescence. The phosphor screen is composed of a quartz substrate coated with the phosphor powder. To make this phosphor screen, MgAl_2_O_4_ powder crystallites of less than 150 µm were selected by using a mesh filter, and its paste was formed by combining the synthesized MgAl_2_O_4_ powder of 1 g with a cellulose binder of 5 mL (EC Vehicle, Nissin Kasei Co., Ltd, Japan). This paste was coated on the quartz substrate using a screen‐printing method using a 50 mesh screen and subsequently fired at 480 °C for 30 min in an air atmosphere to remove the binder. After this procedure, a 100 nm thick Al film was deposited on this anode as a metal back electrode to i) reduce the anode resistivity, ii) reflect the backscattering component of far‐UVC emission frontward, and iii) reduce the buildup potential due to high‐density electron irradiation in vacuo. The size of the device currently used is 45 mm in diameter and 100 mm in length. This size originates from the use of a large phosphor screen (20 mm × 20 mm), readymade glass tubes, and readymade vacuum tube sockets. Therefore, it is possible to reduce the device volume to 1/10 when we use a smaller phosphor screen (10 mm × 10 mm).

### 2.2 Physical Characteristics of the Graphene Nanostructure Cold Cathode

**Figure 2:**
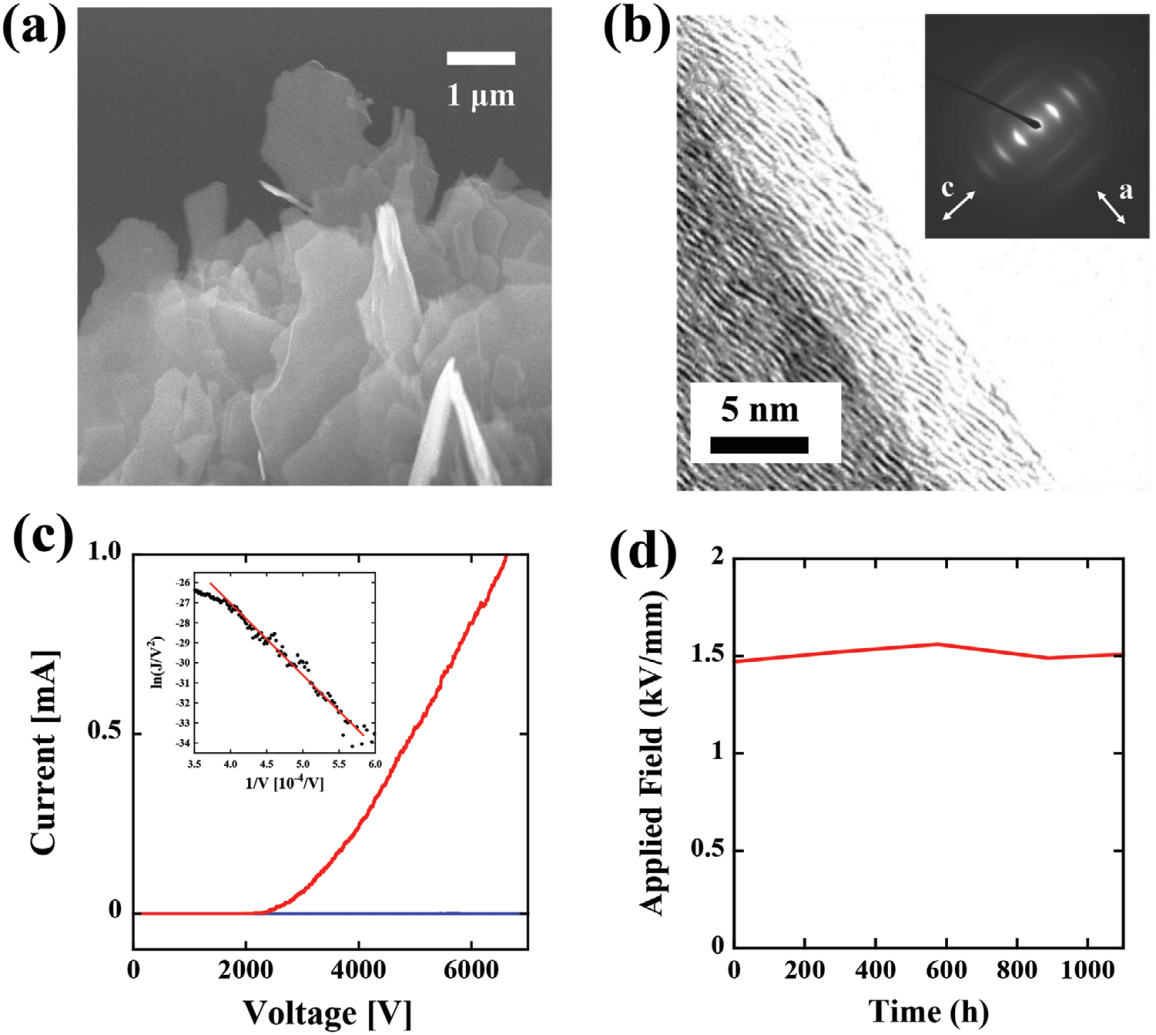
a) SEM image of the microstructure of the GN cold cathode. b) HR‐TEM image of the GN cathode. The inset shows the diffraction patterns. c) Current–voltage (*J*–*V*) characteristics of the GN cathode plotted by the red line. As a comparison, the *J*–*V* characteristics of the bare graphite rod are also plotted by the blue line. The inset shows the Fowler–Nordheim plot for the *J*–*V* characteristics of the GN cathode by setting the horizontal axis as 1/*V* and the vertical axis as ln (*J*/*V* ^2^). d) Lifetime test of the GN cold cathode for 1000 h.

The GN cold cathode was fabricated via plasma etching a carbon rod by a combustion reaction with mixed oxygen and hydrogen gases for 30 s.^[^ ^36^ ^]^ A scanning electron microscope (SEM) image of the microstructure of the GN cold cathode is shown in **Figure** **2**a. Through this etching process, the shape at the edge of the rod was sharpened. The radius of curvature in the top region of the GN was reduced to ≈500 nm, and this thin layer and small radius made the GN suitable as a field emission cathode. A high‐resolution transmission electron microscopy (HR‐TEM) image of the GN cathode is shown in Figure 2b. As indicated by the lattice fringe and diffraction patterns shown in the inset of Figure 2b, the top region of the GN consists of 2D graphene sheets with an interplanar spacing of 0.36 nm.

The field emission properties (current–voltage (*J*–*V*) characteristics) of the GN cathode are plotted in Figure 2c. As a comparison, the *J*–*V* characteristics of the bare graphite rod before etching are also plotted by the blue line. The *J*–*V* characteristics of the GN cathode exhibited an exponential‐like behavior with a high current (red line), whereas the bare graphite rod did not show any electron emission (blue line). The fact that the GN cathode can generate a higher current exceeding 1 mA highlights the possibility of obtaining an intense far‐UVC light source on the order of 150 mW. (The power efficiency of this source is ≈0.6%, and this value will be described in a later section.) According to the Fowler–Nordheim electron emission theory,^[^ ^37^ ^]^ the emission current is described as

(1) $$\begin{array}{c} J=A\left(\frac{\beta^{2}E^{2}}{\varphi }\right)\exp\left(-\frac{B\varphi^{3/2}}{\beta E}\right) \end{array}$$

where *J* is the emission current, *E* = (*V*/*d*) is the applied field, *d* is the distance between the anode and the cathode, *V* is the applied voltage, φ is the work function, which is 4.5 eV for the GN cathode,^[^ ^38^, ^39^ ^]^ *A* and *B* are constants with values of 1.56 × 10^−10^ A V^−2^ eV and 6.87 × 10^9^ V eV^−3/2^ m^−1^, and β is the field enhancement factor. The field enhancement factor (β) can be estimated from the Fowler–Nordheim plot by setting the horizontal axis as 1/*V* and the vertical axis as ln (*J*/*V* ^2^) (natural log), which is shown in the inset of Figure 2c. The slope of the Fowler–Nordheim plot is equal to *Bφ* ^3/2^ *d*/β, and from this relation, we can estimate the field enhancement factor (β) of the GN cathode to be 7914. This value is one of the largest values ever reported for carbon emitters;^[^ ^40^, ^41^ ^]^ thus, the GN cathode is suitable for high‐current operation.

The stability and durability of the emission are quite important for practical use. Figure 2d shows the lifetime test results of the GN cathode conducted at a constant current of 500 µA and a base pressure of 10^−7^ Pa. We observed no degradation of the anode applied field in maintaining a constant current, and the lifetime of the GN exceeded 1000 h. The lifetime testing of the GN cathodes is now in progress. It is generally known that ion bombardment causes the degradation of the cathode and the bombardment rate is correlated with the base pressure.^[^ ^42^, ^43^ ^]^ Therefore, we consider that the lifetime of the GN cathodes can be exceeded to more than 10 000 h by reducing the base pressure below 10^−8^ Pa.

### 2.3 Physical Characteristics of the MgAl_2_O_4_ Phosphor Anode

**Figure 3:**
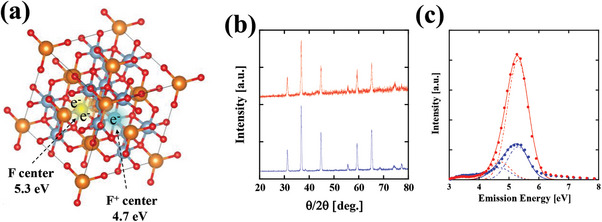
a) Images of the F‐center (yellow cloud) and F^+^‐center (green cloud) wavefunctions at the oxygen‐removed defect states. The defect states are filled with two (F‐centers, 5.3 eV) or one (F^+^‐centers, 4.7 eV) electron. b) X‐ray diffraction patterns of the synthesized phosphor (red line) and a reference MgAl_2_O_4_ spinel crystalline powder (blue line). c) CL spectrum of the synthesized MgAl_2_O_4_ phosphors (red circles) and that of the reference MgAl_2_O_4_ powder (blue circles) with the emission peak at 5.3 eV (230 nm) and full width at half maximum (FWHM) of 0.8 eV (30 nm). Here, the CL spectra were obtained by a 50 µA total current‐accelerated 25 kV electron beam. The experimentally observed CL spectra were fitted by two Gaussian functions with the peak energies of the F‐centers and F^+^‐centers. Both Gaussian functions are assumed to have the same energy width of 0.5 eV (FWHM). For the synthesized phosphor, the height of the F‐center is fitted by 2000 (red broken line) and that of the F^+^‐center is fitted by 300 (red dotted line). For the reference powder, the height of the F‐center is fitted by 550 (blue broken line) and that of the F^+^‐center is fitted by 300 (blue dotted line). The solid red and blue lines represent the sums of the theoretical F‐ and F^+^‐center curves.

As far‐UVC‐emitting phosphors, direct‐transition wide‐bandgap semiconductors are suitable for electron beam excitation because of their high luminous efficiency. MgAl_2_O_4_ is one of the materials proposed for use as a far‐UVC phosphor. The bandgap of MgAl_2_O_4_ was previously reported to be 7.8 eV, which corresponds to emission at 159 nm.^[^ ^44^, ^45^, ^46^ ^]^ This bandgap emission originating from an interband transition cannot propagate in an air atmosphere because air itself absorbs the emission. However, the introduction of defects and oxygen vacancies, which are known as F‐centers,^[^ ^47^ ^]^ in the MgAl_2_O_4_ lattice can result in strong luminescence in the far‐UVC region at ≈5.3 eV (230 nm).^[^ ^48^, ^49^, ^50^ ^]^ Thus, the emissions from these F‐centers are suitable to be applied as sterilization radiation in a safe manner. Removing an oxygen atom from the MgAl_2_O_4_ lattice results in s‐like wavefunction defect states, as shown in **Figure** **3**a, that can be filled with two (F‐centers, 5.3 eV) or one (F^+^‐center, 4.7 eV) electron. In this work, defects and oxygen vacancies were introduced via the synthesis temperature. For example, MgAl_2_O_4_ powder was synthesized in a solid‐phase reaction. First, 99.99% purity magnesium oxide (MgO) and alumina (α‐Al_2_O_3_) powders (high‐purity materials, Kojundo Chemical Laboratory Co., Ltd) were mixed well and fired at 1200 °C for 3 h in an oxygen atmosphere, where the mole ratio of MgO to α‐Al_2_O_3_ was maintained at 1 to 1. Figure 3b shows the X‐ray diffraction patterns (red line) acquired from the synthesized phosphor at 1200 °C. As a reference, the X‐ray diffraction patterns of a commercially available MgAl_2_O_4_ powder (high‐purity materials, Kojundo Chemical Laboratory Co., Ltd, Japan) are also shown by the blue line. Although the peak intensities differ for the two samples, both diffraction peaks are situated at the same angles. These diffraction patterns show that both the synthesized and commercially available MgAl_2_O_4_ powders have the same spinel crystal structures.

Figure 3c shows the CL spectrum of the synthesized MgAl_2_O_4_ phosphors (red circles). We observed the CL spectrum with the emission peak at 5.3 eV (230 nm) and an FWHM of 0.8 eV (30 nm). As a reference, the CL spectrum of a commercially available MgAl_2_O_4_ powder is also shown by blue circles. Here, the CL spectra were obtained by a 50 µA total current‐accelerated 25 kV electron beam. The peak intensity of the synthesized phosphor was more than three times higher than that of the reference powder. In addition, the unnecessary luminescence in the range from 3 to 4 eV (from 300 to 400 nm) was inhibited, and the obtained power efficiency of the phosphors was ≈0.6%. This efficiency is low. A simple way to improve the CL efficiency is the fabrication of a textured structure on the backside of the quartz substrate. By applying this approach, the CL extraction efficiency can be improved by a factor of 2–3. Because, all CLs with an incident angle larger than the critical angle (≈40° by using the refractive index at the far‐UVC region as 1.53)^[^ ^51^, ^52^ ^]^ are reflected and trapped inside the quartz substrate.

The spectra were originally measured as a function of wavelength and CL intensity. However, the CL spectra in Figure 3c were converted from the wavelength domain into the energy domain to clearly show the distinction between the F and F^+^ centers. Note that a λ^2^ correction must be applied when we convert wavelength into energy. The CL spectrum was fitted by a Gaussian function as

(2) $$I\left(\varepsilon \right)=\alpha \exp\left[-{\left(\frac{\varepsilon -\xi }{\Delta \varepsilon }\right)}^{2}\right]$$

where *I*(ε) is the F‐ or F^+^‐center spectrum as a function of energy ε, α is the height of the curve's peak, Δε is the width of the curve (FWHM), and ξ is the position of the center of the peak. The F‐center spectrum can be fitted by ξ = 5.4 eV, and the F^+^‐center spectrum can be fitted by ξ = 4.7 eV, with an energy width of Δε = 0.5 eV for both spectra.^[^ ^48^, ^50^ ^]^ By fitting the experimentally observed CL spectra using these curves, we can determine the ratio between the F‐center and F^+^‐center spectra. For the synthesized phosphor, the height of the F‐center is well fitted by α_SP_^F^ = 2000 (red broken line) and that of the F^+^‐center is well fitted by α_SP_^F+^ = 300 (red dotted line). On the other hand, for the reference powder, the height of the F‐center is fitted by α_RP_^F^ = 550 (blue broken line) and that of the F^+^‐center is fitted by α_RP_^F+^ = 300 (blue dotted line). This result emphasizes that our synthesized phosphor efficiently emits the required spectral region (larger than 5.3 eV) with the portion exceeding 50% and that the phosphor strongly emits the F‐center band more than three times compared to the reference powder. These facts suggest that the introduction of F‐center vacancies was successfully achieved by our synthesized method.

### 2.4 Germicidal Efficacy of *Escherichia coli* by the Far‐UVC Light Source

We performed germicidal experiments to study the germicidal efficacy of our far‐UVC devices. A pure culture of *E. coli* was incubated in nutrient broth (E‐MC63; EIKEN Chemical Co., Tokyo, Japan) at 37 °C for 20 h. Bacteria at a concentration of 10^9^–10^10^ colony‐forming units (CFU) mL^−1^ were grown and used for the experiments. Then, the *E. coli* culture solution was diluted to a concentration of 10^4^–10^5^ CFU mL^−1^ using a normal saline solution (0.9 g NaCl dissolved in 100 mL purified water) to precisely measure the efficacy. Then, 500 µL of the diluted culture solution was added to a multiwell plate (24 wells). This well plate was then placed 5 cm away from the far‐UVC light source. The far‐UVC dose conditions used were 4, 8, and 12 mJ cm^−2^, where the irradiance used was 40 µW cm^−2^. Here, the irradiance was measured by a UV‐extended Si photodiode (S120VC, Thorlabs Inc., New Jersey, USA).

**Figure 4:**
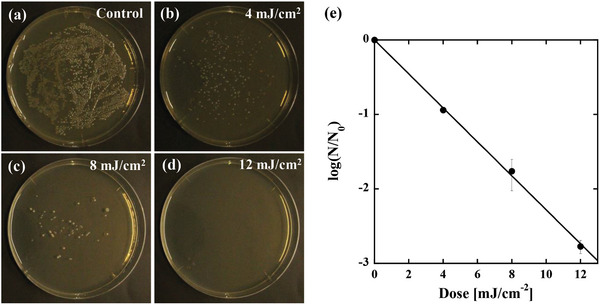
Germicidal efficacy achieved by our fabricated far‐ultraviolet C (UVC) devices. A pure culture of *E. coli* was used to investigate the germicidal efficacy. a) Control plate (0 mJ cm^−2^ dose). b) Plate disinfected with a 4.0 mJ cm^−2^ dose. c) Plate disinfected with an 8.0 mJ cm^−2^ dose. d) Plate disinfected with a 12.0 mJ cm^−2^ dose. e) Log‐reduction behavior (normal log) as a function of the dose. The inactivation rate constant can be fitted by the slope of the log‐reduction behavior, which results in a value of κ = 0.58 cm^2^ mJ^−1^.

The results of the efficacy of disinfection by using the far‐UVC light source are shown in the photographs of **Figure** **4**a–d, where Figure 4a is the control plate (0 mJ cm^−2^ dose), Figure 4b is the plate disinfected by a 4.0 mJ cm^−2^ dose, Figure 4c is the plate disinfected by an 8.0 mJ cm^−2^ dose, and Figure 4d is the plate disinfected by a 12.0 mJ cm^−2^ dose. For counting CFUs in the range of 10^1^–10^4^, we took a digital image of the plate (10^1^–10^3^) or diluted plate (CFUs > 10^3^), where we used Processing software (https://processing.org/) for the calculation of CFUs.

The number of colonies decreased significantly with increasing irradiation dose. To quantitatively investigate the reduction in inactivation rates as a function of the dose, we plotted the obtained CFU responses as shown in Figure 4e. Here, all experimental results are reported as the means across three replicates. The disinfection kinetics as a function of the dose were determined by

(3) $$\begin{array}{c} \ln\left(\frac{N\left(D\right)}{N_{0}}\right)=-\kappa D \end{array}$$

where κ (cm^2^ mJ^−1^) is the natural log dose‐based disinfection rate constant, *D* is the magnitude of the far‐UVC dose (mJ cm^−2^), *N*_0_ is the number of CFUs on the un‐irradiated control (CFU mL^−1^), and *N*(*D*) is the number of CFUs at a given dose *D*. The far‐UVC dose–response results can be fitted using a single disinfection constant, κ = 0.58 (cm^2^ mJ^−1^), as shown by the solid line in Figure 4e. This value is smaller than the previously observed value of κ = 1.2 (cm^2^ mJ^−1^) at 220 nm.^[^ ^53^ ^]^ We consider that this difference can be elucidated by the overlap between the action spectra of *E. coli* ^[^ ^53^ ^]^ and the energy width of the far‐UVC light sources. For example, a broad CL spectrum ranging from 4 to 6 eV was used in this study, whereas the narrow single spectrum (5.64 eV) transmitted through the interference filter was used in a previous study. The action spectrum has a peak at 5.64 eV. Therefore, the broad CL spectrum shows a smaller disinfection rate constant than that obtained by the efficient disinfection band at 5.64 eV. The quantitative evaluation of the rate constant is an issue to be solved in the future.

## 3 Discussion

**Figure 5:**
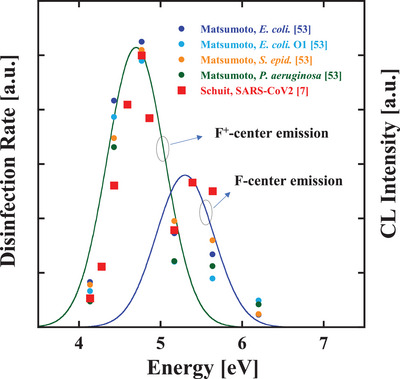
Superposition of F^+^‐center emission spectrum (green line: the peak energy of 4.7 eV and an FWHM of 0.5 eV), F‐center emission (blue line: the peak energy of 5.3 eV and an FWHM of 0.5 eV), and the previously reported disinfection rate constants of *E. coli* DH5α (blue circles), *E. coli* O1 (light blue circles), *Pseudomonas aeruginosa* (green circles), *Staphylococcus epidermidis* (orange circles) obtained by Matsumoto et al.,^[^ ^53^ ^]^ and SARS‐CoV‐2 (red squares) obtained by Schuit et al.^[^ ^7^ ^]^

When we superimpose the obtained CL emission on the previously reported action spectra of various bacteria^[^ ^53^ ^]^ and SARS‐Cov2,^[^ ^7^ ^]^ it is possible to show that the MgAl_2_O_4_ phosphors presented here are suitable for disinfecting various bacteria and viruses. **Figure** **5** shows the action spectra composed of the disinfection rate constants of *E. coli* DH5α (blue circles), *E. coli* O1 (light blue circles), *Pseudomonas aeruginosa* (green circles), *Staphylococcus epidermidis* (orange circles) obtained by Matsumoto et al.,^[^ ^53^ ^]^ and SARS‐CoV‐2 (red squares) obtained by Schuit et al.^[^ ^7^ ^]^ Below 5.2 eV, the same action spectrum is found for these bacteria and SARS‐CoV‐2. Notably, the fact that the peak of the F^+^‐center emission (4.7 eV = 265 nm) described by the green curve of Figure 5 precisely coincide with the peak of the action spectra suggests that the F^+^‐center emission is suitable for the disinfection of both bacteria and viruses. On the other hand, above 5.2 eV, SARS‐CoV‐2 shows higher disinfection rates compared to those of the bacteria. (The reason for this difference is not clearly understood; however, we consider that the difference of the disinfection rates above 5.2 eV originates from the thickness of the protein layer, which covers DNA or RNA genome (the difference of the absorbance).). The F‐center emission (5.3 eV = 230 nm) described by the blue curve of Figure 5 precisely coincides with the second peak of the action spectra of SARS‐CoV‐2. This fact suggests that the F‐center emission is suitable for the disinfection of the viruses in a safe manner. Selecting and narrowing the F‐center and/or F^+^‐center spectra are issues to be solved, and we will discuss these points as follows.

As shown in Figure 3c, for the F‐center and F^+^‐center bands, both the synthesized phosphor and the reference powder have the same spectral width of 0.5 eV. Therefore, it seems to be difficult to make a narrower spectrum by making higher‐quality MgAl_2_O_4_ crystals. Because the broad spectrum originates from the strong coupling between electron wavefunctions and localized lattice vibrations, thus the broad spectrum itself is intrinsic. To solve this issue, we are currently planning to obtain stimulated emission from the F‐center band. In this regard, the graphene nanostructure field emitter seems to be promising because this cathode can achieve an extremely high current exceeding 1 mA as well as a high brightness exceeding 10^11^–10^12^ A sr^−1^ m^−2^.^[^ ^54^ ^]^ These features are suitable for performing high current density excitation, leading to obtain stimulated emission from these centers. Achieving spectral narrowing by exciting a high‐density electron beam is currently under investigation and will be reported elsewhere.

Based on the discussion in the literature about energy loss processes of CL,^[^ ^55^ ^]^ it is necessary to consider the following losses: i) reflection of the incident electron beam (Tomlin's relation);^[^ ^56^ ^]^ ii) the minimum ionization energy of the bandgap, which is correlated with the stopping power;^[^ ^57^ ^]^ and iii) the Stokes shift between the bandgaps of the MgAl_2_O_3_ and F‐centers.^[^ ^58^ ^]^ By considering these loss factors, the total theoretical efficiency of CL of MgAl_2_O_4_ phosphors can be estimated as 25%, assuming that the light extraction efficiency is 100%. However, at present, the obtained CL efficiency is low on the order of 0.6%. We consider that the low efficiency originates from the small number of F‐centers formed in the MgAl_2_O_3_ phosphors. To increase the number of F‐centers, we are currently re‐examining the stoichiometry between Al_2_O_3_ and MgO. (It should be noted here that the increase of the efficiency with the spectral shift toward shorter wavelength region is obtained by the phosphors with a higher MgO molar ratio.) The determination of the optimal stoichiometry and a lifetime testing of the phosphors are currently under investigation and will be reported elsewhere.

**Figure 6:**
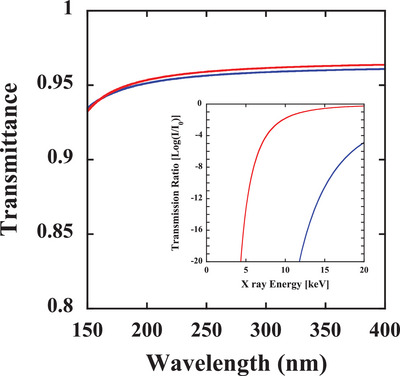
Optical transmittance of the fused silica (red line) and BaF_2_ (blue line) substrates in the far‐UVC wavelength region. The inset shows the X‐ray transmission curve versus energy for the fused silica (red line) and BaF_2_ (blue line) substrates with a thickness of 1 mm.

Note that when using higher‐energy electron beams of the order of 10 keV to obtain a far‐UVC emission, bremsstrahlung X‐rays are also generated as well.^[^ ^59^ ^]^ To fabricate a far‐UVC light source that is safe for human body, it is necessary to shield the emission of these bremsstrahlung X‐rays inside the far‐UVC light source. As a transparent window material for the far‐UVC wavelength region, fluoride substrates, such as BaF_2_, and fused silica substrates can be considered. **Figure** **6** shows the transmittance of both fused silica (red line) and BaF_2_ (blue line) substrates in the far‐UVC wavelength region. Here, the transmittance was calculated using the Fresnel equation,^[^ ^60^ ^]^ where far‐UVC light is assumed to enter perpendicular to the substrate, and the wavelength dispersion of the refractive index of the fused silica is based on refs. [51, 52] and that of BaF_2_ is based on ref. [61]. Both substrates show higher transmittance than 0.95 in the far‐UVC wavelength region, as shown in Figure 6. However, due to the large difference in density, 2.2 g cm^−3^ for fused silica^[^ ^60^ ^]^ and 4.9 g cm^−3^ for BaF_2_,^[^ ^62^ ^]^ the transmission curve of X‐rays significantly differs with respect to the energy. The inset of Figure 6 shows the transmission curve versus energy for the fused silica (red line) and BaF_2_ (blue line) substrates with a thickness of 1 mm. When we used 7 keV energy electron beams to obtain far‐UVC CL, the maximum bremsstrahlung X‐ray energy became 7 keV. In this case, both substrates can shield (absorb) the generated X‐ray inside the vacuum tube. However, if we apply a higher energy for the acceleration of electron beams more than 7 keV, the shield ability of the fused silica for the generated X‐ray is drastically weakened. Therefore, a higher‐density material such as BaF_2_ is suitable for shielding the generated X‐ray. However, BaF_2_ is designated as a deleterious and poisonous substance; therefore, to make a practical far‐UVC light source using CL, the acceleration voltage must be kept as low as possible to avoid the transmission of bremsstrahlung X‐rays from the fused silica window.

## 4 Conclusion

In summary, we have demonstrated the fabrication of solid‐state far‐UVC light sources using graphene nanostructure field emitters and wide‐bandgap MgAl_2_O_4_ phosphor materials for the first time. The lifetime of the light sources exceeds 1000 h. The lifetime test of the far‐UVC light sources with a higher vacuum degree than 10^−7^ Pa is ongoing, and the result is expected to be longer than 10 000 h. By using the far‐UVC light sources presented herein, we have also demonstrated the efficient disinfection of *E. coli*. We believe that the future developed solid‐state far‐UVC light sources based on the graphene nanostructure and wide‐bandgap phosphor will facilitate future technologies for preventing the spread of infectious diseases in a safe and convenient manner.

## Conflict of Interest

The authors declare no conflict of interest.

## Author Contributions

Y.N. is the first author. T.M. conceived the idea and supervised the work at all stages. Y.N. and G.H. contributed to the measurements of the field emission characteristics. G.H., R.K., and T.O. synthesized the magnesium aluminate phosphors. Y.N. and R.K. performed screen printing of the phosphors. Y.N., T.O., and T.M. contributed to measuring and analyzing the crystal structures and optical characteristics of the magnesium aluminate phosphors. G.H. and R.K. performed the *E. coli* disinfection experiments. All authors read and approved this submitted manuscript.

## Data Availability

The data that support the findings of this study are available from the corresponding author upon reasonable request.
